# A machine learning tool for the diagnosis of SARS‐CoV‐2 infection from hemogram parameters

**DOI:** 10.1111/jcmm.17864

**Published:** 2023-10-26

**Authors:** S. Gómez‐Rojas, G. Pérez Segura, J. Ollé, G. Carreño Gómez‐Tarragona, J. González Medina, J. M. Aguado, E. Vera Guerrero, M. Poza Santaella, J. Martínez‐López

**Affiliations:** ^1^ Department of Hematology Hospital Universitario 12 octubre Madrid Spain; ^2^ Conceptos Claros Co Barcelona Spain; ^3^ Department of Hematology Hospital Universitario Fundación Jiménez Díaz Madrid Spain; ^4^ Unit of Infectious Diseases Hospital Universitario "12 de Octubre", Instituto de Investigación Sanitaria Hospital "12 de Octubre" (i+12), CIBERINFEC, ISCIII Madrid Spain; ^5^ Department of Medicine, School of Medicine Universidad Complutense Madrid Spain

**Keywords:** cell morphological data, cell population data, COVID‐19, hemogram, machine learning, SARS‐CoV‐2

## Abstract

Monocytes and neutrophils play key roles in the cytokine storm triggered by SARS‐CoV‐2 infection, which changes their conformation and function. These changes are detectable at the cellular and molecular level and may be different to what is observed in other respiratory infections. Here, we applied machine learning (ML) to develop and validate an algorithm to diagnose COVID‐19 using blood parameters. In this retrospective single‐center study, 49 hemogram parameters from 12,321 patients with clinical suspicion of COVID‐19 and tested by RT‐PCR (4239 positive and 8082 negative) were analysed. The dataset was randomly divided into training and validation sets. Blood cell parameters and patient age were used to construct the predictive model with the support vector machine (SVM) tool. The model constructed from the training set (5936 patients) achieved an accuracy for diagnosis of SARS‐CoV‐2 infection of 0.952 (95% CI: 0.875–0.892). Test sensitivity and specificity was 0.868 and 0.899, respectively, with a positive (PPV) and negative (NPV) predictive value of 0.896 and 0.872, respectively (prevalence 0.50). The validation set model (4964 patients) achieved an accuracy of 0.894 (95% CI: 0.883–0.903). Test sensitivity and specificity was 0.8922 and 0.8951, respectively, with a positive (PPV) and negative (NPV) predictive value of 0.817 and 0.94, respectively (prevalence 0.34). The area under the receiver operating characteristic curve was 0.952 for the algorithm performance. This algorithm may allow to rule out COVID‐19 diagnosis with 94% of probability. This represents a great advance for early diagnostic orientation and guiding clinical decisions.

## 1 INTRODUCTION

Machine learning (ML) tools constitute a method for data analysis that automate the construction of analytic models. This is because systems can learn from data, identify patterns and make decisions with minimal human intervention. In this sense, its application is increasing in biomedical research, engaging health researchers in a process of discovery around developing data‐driven algorithms to make clinically reliable predictions.^1^, ^2^, ^3^ There are several studies in the haematology field that use ML tools for guiding diagnosis of haematological diseases^4^, ^5^, ^6^ and they have been successfully applied to general image recognitions, including histopathological images, to assist the process of medical diagnosis.^7^, ^8^, ^9^, ^10^

Shouval et al. analysed data of patients in the European Society for Blood and Marrow Transplantation and succeeded in constructing a prediction model of overall mortality after.^11^ Other studies have reported on applications of ML for the prediction of relapse risk and for stratification of early‐stage haematological disease.^12^, ^13^, ^14^ For instance, Pan et al. successfully applied ML for the identification of prognostic factors of childhood acute lymphoblastic leukaemia based on medical data.^15^ For the most part, ML algorithms have been investigated for hematologic diagnosis using specific laboratory, histopathology, flow cytometry and molecular data, and very few studies have used laboratory data.^4^, ^16^, ^17^, ^18^

Cellular and molecular changes caused by several diseases are directly or indirectly usually detectable through modifications in blood parameters, provided by new more refined haematology automatized analyzers. From this basis, few studies have been carried out, with application of different prediction models including identification of blasts and different subtypes of acute leukaemias. Some of them have developed analytic and tree decision models for blasts detection from cell morphological data, more concretely in distinction of acute promyelocytic leukaemia.^19^

Currently, in addition to usual hemogram parameters, automatized analyzers provide even more information, turning cellular morphology changes into numeric and objective information. The Beckman Coulter DXH 900 technology is based on cellular volume, conductivity, and laser scatter, providing information of leucocyte subpopulation and cell morphologic data (CMD). These CMD are numerical data that reflect different morphological features of the leucocytes such as size, cytoplasm complexity, nucleus/cytoplasm ratio, granularity etc. Moreover, this analyser offers a new parameter: monocyte distribution width (MDW), defined as an early sepsis indicator (ESI).^20^, ^21^, ^22^

It is well established that the hyperinflammatory response induced by SARS‐CoV‐2 is the major cause of disease severity and monocytes play the main role in cytokine storm, changing their conformation, function and phenotype. However, other studies also suggest that neutrophils may also have a key role in the disease pathophysiology. According to the literature, those changes may be different in SARS‐CoV‐2 infection other respiratory infections such as virus influenza.^23^, ^24^ COVID‐19 pandemic has globally exceeded health systems, and has laid bare the need for improved diagnostic tools to monitor/control SARS‐CoV‐2 infection. While microbiological testing based on reverse transcription (RT)‐PCR remains the gold standard, simple, reliable and inexpensive tests that could help in diagnosing SARS‐CoV‐2 infection.^24^ In the present study, we sought to explore the possibility of generating a decision‐making ML algorithm to allow the classification and prediction of COVID‐19 diagnosis based on blood parameters. Our primary goal was to generate an algorithm by ML tools for the accurate diagnosis of SARS‐CoV2 infection in an efficient and early manner in patients with respiratory symptoms. As a secondary goal, we evaluated the accuracy of the algorithm in patients at different clinical stages, including critical patients.

## 2 MATERIALS AND METHODS

### 2.1 Study setting and population

The present study is a retrospective single‐center study performed between January 2020 and March 2021. Data were collected from Hospital Universitario 12 de Octubre (H12O), a Spanish tertiary referral center. All enrolled patients (*N* = 12,321) had been admitted in the Emergency Department with respiratory symptoms and were tested for RT‐PCR, resulting in 4239 positive patients and 8082 negative patients. RT‐PCR was performed using the GeneXpert® analyser (Cepheid). From the 8082 negative patients we study a subgroup of patients that have a positive RT‐PCR test for influenza A and B virus (*N* = 81).

We excluded paediatric patients (<18 years) and patients with haematological malignancies. We collected data of age (median 72 years) and gender (64% males, 36% females).

Blood samples were processed within 2 h of extraction The specimens were contained in tubes with EDTA 2k. In total 48 parameters from hemogram were analysed and, together with the patients age, had been used to construct the analytic model.

**FIGURE 1:**
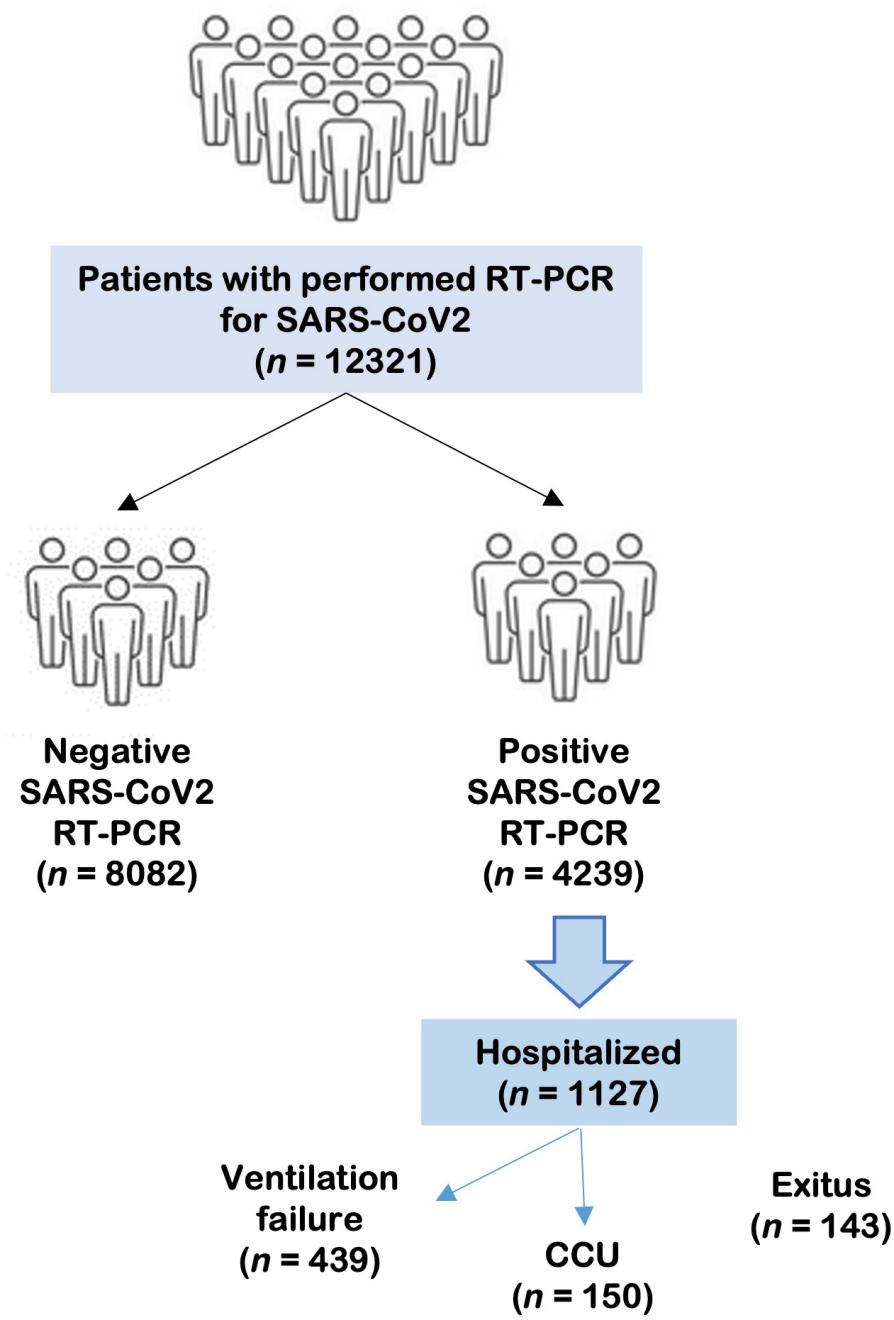
Flow chart shows the distribution of the patients included in the study, a total of 12,321. Patients with infectious or respiratory symptons are divided in a negative or positive group according to PCR result. In the subgroup of hospitalized patients, patients are classified based on clinical severity: ventilatory failure, admitted to critical care unit (CCU) or exitus.

In a second step, clinical data from a subgroup of hospitalized patients 1127 were collected: ventilatory failure (VF) (defined as invasive mechanical ventilation required), exitus (E), and admission to critical care unit (CCU). A total of 439 hospitalized patients had VF, 150 were admitted to CCU and 143 patients dead caused of the COVID‐19 (Figure 1).

This project was included in the H12O ImmunoCovid study and the H12O ethical committee approved it.

### 2.2 Statistical analysis and ML algorithm generation

The prediction model was developed using the R studio Software and the CARET (Classification and Regression Training) package. Database was divided into two random equal subgroups: the training group to train the model, and the validation group to check the results. Then, these groups were balanced by down sampling strategy, remaining a total of 5936 patients in the training group (half of them with COVID‐19 positive diagnosis and the other half with negative) and 4964 in the validation one (a total of 10,900 patients). We used a supervised strategy. The best algorithm evaluated was obtained by supervised support vector machines (SVM) tool.

SVM is a supervised learning algorithm that get the characteristics of known items in multiple dimensions and then build predictive models to classify data of unknown classification.^25^

In our study, each patient expressed different values of blood test parameters, so the distributions are separated in a multidimensional space. Consequently, when data from SARS‐CoV‐2 infected and non‐infected patients are mixed to make up the training data file, the dividing plane between both groups differed in the multidimensional space. Thus, finding the corresponding optimal parameters of the individual‐specific: C parameter (C) and sigma (γ) in this dividing plane was the key to establish the best SVM model.^32^ It was decided to choose the SVM model by presenting a median of the area under the ROC curve of 0.88. No data preprocessing was performed, and the parameters chosen for the model were: *γ* = 0.014; C = 1; and Number of Support Vectors = 458. The evaluation of the different models was based on the comparison of the efficacy obtained through a 10‐fold cross‐validation.

We used 49 parameters, including total white blood cells (WBC): total leucocytes, neutrophils, lymphocytes, monocytes, 41 parameters of CMD from neutrophils, monocytes and lymphocytes, WBC differential optical count (IWDOP), MDW, immature granulocytic cells (IEGC), and age as the unique clinical variable, cause it may influence morphological changes in circulating cells.

The performance of the pattern in this model was evaluated using receiver operating characteristic (ROC) curve. The sensitivity, specificity, accuracy, positive predictive value (PPV) and negative predictive value (NPV) were also computed by confusion matrix‐derived metrics.

With respect of the model performance in different clinical groups, we assessed differences in the accuracy of the model based on the severity of the patient. The evaluation of differences was performed by *t*‐test.

## 3 RESULTS

### 3.1 Machine learning algorithm generation

**FIGURE 2:**
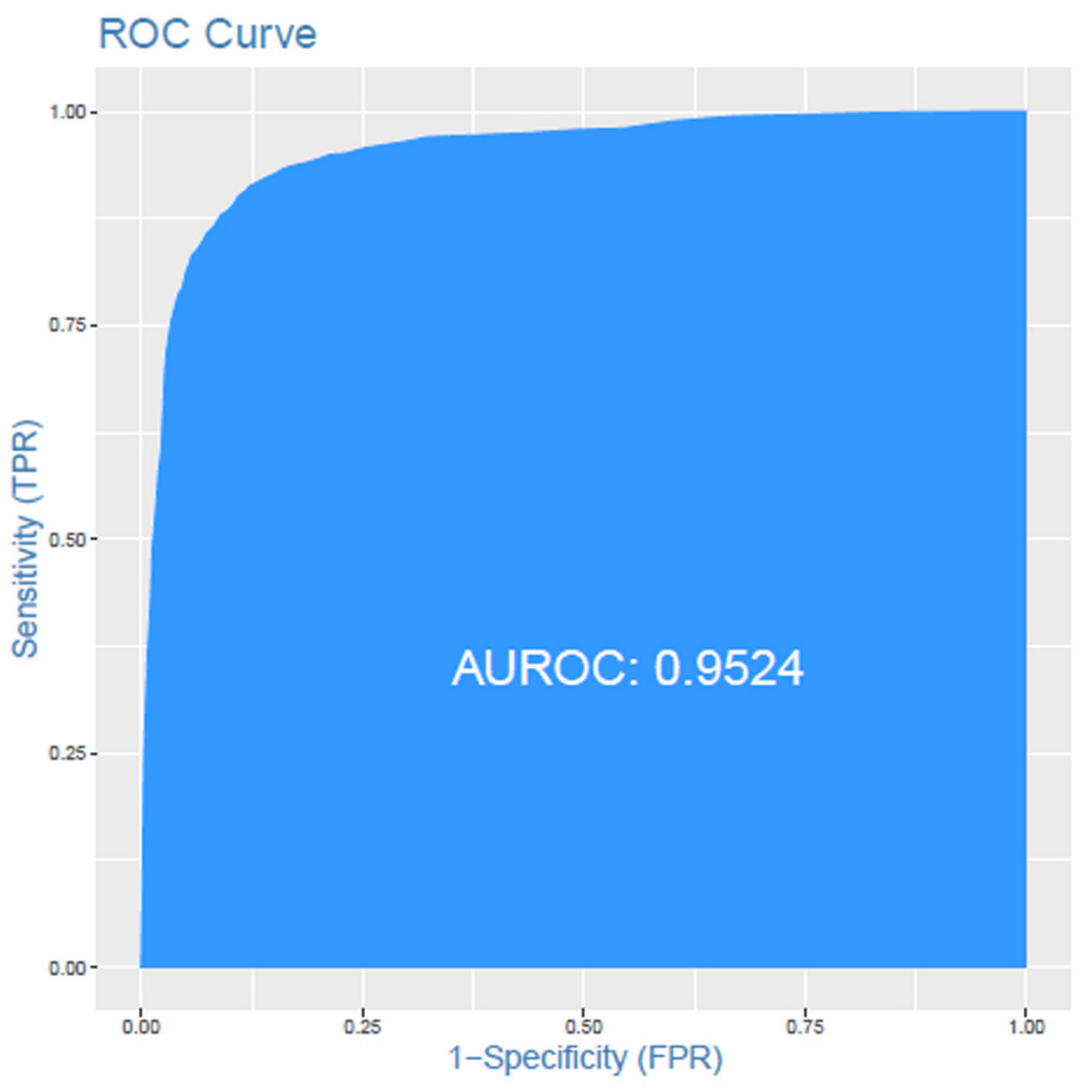
ROC curve: Results from the validation test. The accuracy for COVID‐19 infection prediction with an AUC of 0.9524.

Using SVM we generate a predictive model trained with a total of 5936 patients tested (2968 positive COVID‐19 RT‐PCR and 2968 negative COVID‐19 RT‐PCR). The accuracy for the diagnosis of SARS‐CoV2 infection in this training phase was 0.852 (95% CI: 0.875–0.892). The test sensitivity and specificity were 0.868 and 0.899 respectively with a PPV: 0.896 and NPP: 0.872 (prevalence: 0.50). Results obtained from the validation test phase, applied to 1271 COVID‐19‐positive patients and 3693 COVID‐19‐negative patients (total of 4964 patients), were the following: accuracy 0.894 (95% CI: 0.883–0.903), sensitivity 0.8922 and specificity 0.8951 with PPV 0.817 and NPV: 0.94 (prevalence: 0.344). Table 1 The ROC curve showed an area under curve (AUC) of 0.952 for this classification algorithm model (Figure 2).

**TABLE 1:** Contingency table. Results from validation phase.

Algorithm results		
	Real diagnosis	
Prediction	COVID+	COVID−
COVID	454	704
No COVID	79	4436

### 3.2 Accuracy of the model in different clinical groups

According to the different clinical groups we obtained these results (Table 2): the accuracy for the COVID‐19 diagnosis reached 91.87% in hospitalized patients vs non hospitalized group, that was 88% (*p* = 0.0003), 95% CI (1847%–5736%). The accuracy of prediction was 93.48% for patients with VF versus 90.85% in the non‐VF group (*p* = 0.14). Similar results were achieved in patients admitted to CCU with an accuracy of 96.88% and non‐admitted patients to CCU: 91.12% (*p* = 0.024). No differences were found between the exitus group (92.59%) and the non‐exitus group (92%).

**TABLE 2:** Accuracy of the model in different clinical groups.

Data	Accuracy training (%)	Accuracy test (%)	Significance
All patients (*N* = 4964)	88.43	**89.41**	**–**
Non‐hospitalized (*n* = 3837)	88.3	**88**	***p* = 0.0003**
Hospitalized (*n* = 1127)	91.3	**91.87**	
RF=No (*n* = 688)	91.81	**90.85**	*p* = 0.14
RF=Yes (*n* = 439)	92.66	**93.48**	
CCU=No (*n* = 977)	91.67	**91.12**	***p* = 0.024**
CCU=Yes (*n* = 150)	95.88	**96.88**	
E = No (*n* = 974)	93.21	**92**	*p* = 0.92
E = Yes (*n*= 153)	88.35	**92.59**	

*Note:* Bold values are highlighting the statistically significant results.

Abbreviations: CCU, admission in critical care unit; E, exitus; RF, respiratory failure.

**FIGURE 3:**
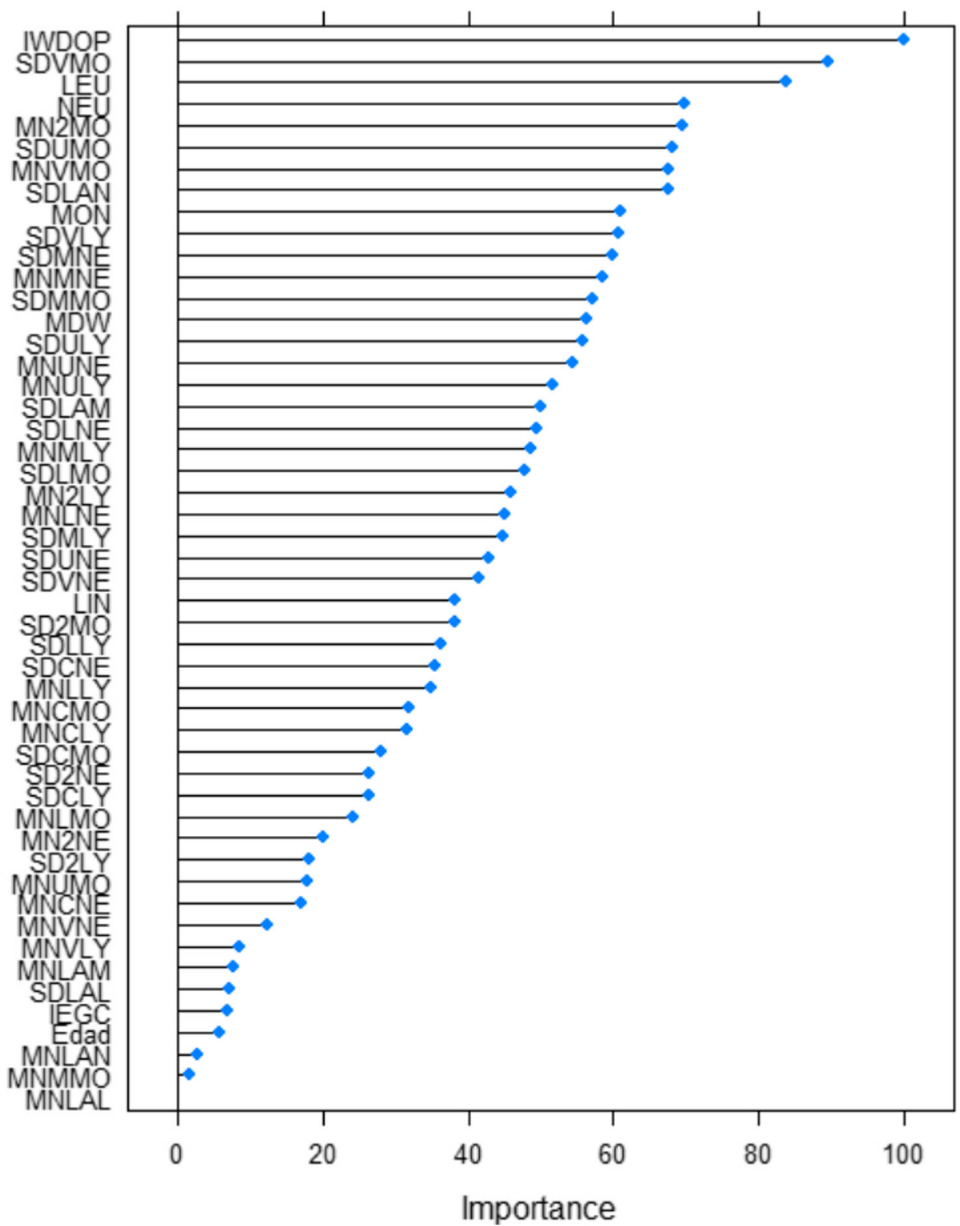
Lists the most important blood parameters (as estimated by ReliefF) and their frequencies. Abbreviations are indicated in Appendix S1.

The importance of each variable in isolation for the development of the algorithm is reported in Figure 3. IWDOP turned out to be the most valuable variable on its own, which corresponds to the leucocyte optical count. The parameters based on monocyte and neutrophil light scattering showed the most importance for the model. Only three parameters without importance by their own were detected. Nonetheless, none of the variables could be removed because the combination and mutual information of all parameters are what make possible to achieve the best accuracy of the algorithm (Figure 3).

### 3.3 Accuracy of the model in differentiation of COVID‐19 infection versus flu

For a subgroup of patients that included 81 patients with flu versus 81 patients with SARS‐Cov 2 infection, the results obtained: a ROC curve with an area of 0.892, with a sensitivity and specificity of 80% and 85%, respectively. A total of 12.96% of false positives for COVID‐19 on the flu group, and 9.72% of false positives for flu on the group of COVID‐19.

## 4 DISCUSSION

ML tools have allowed us to generate an algorithm based on hemogram parameters that is able to diagnose COVID‐19 disease with high accuracy.

### 4.1 Diagnosis of COVID‐19 is complex

Transcriptase polymerase chain reaction (RT‐PCR) has routinely been used to confirm diagnosis of SARS‐CoV2 infection and have been established as the ‘gold standard’. However, diagnostic uncertainties and controversies have arisen. Several authors have pointed out the poor performance of this technique, particularly in terms of sensitivity.^24^, ^25^, ^26^ Important variations in the sensitivity occur according to the different types of collected specimens^27^ and depending on the time of evolution of the disease. Another drawback of RT‐PCR is the requirement of at least 4 h of processing performed by skilled technicians. Antigen detection is another available diagnostic tool for SARS‐CoV2 and has the advantage of the earliness and the lack of the precarious sensibility reported in many studies.^24^, ^25^ It is proposed that the combination of these two techniques should be stablished as gold standard.^24^

These facts led us to search for rapid and accurate tests for SARS‐CoV2 screening, based on routine biological tests. So, we highlight the importance of hemogram not only as a quick screening of haematological disease but also as a basic laboratory tool with and easy and quick performance from which we can get a large amount of information from circulating cells, including useful data for discriminating infection diseases. Evidence of this is the novel ESI, a new name for MDW, based in monocytes morphological changes that improves the sepsis detection with a high S and E.^20^, ^21^ In addition to MDW, novel analyzers offer investigational parameters (CMDs), that attend to cellular conformation and allow the detection of morphological changes that are not detectable by human visualization.^28^ ML is a current tool that can handle a great number of parameters from individual cells to be simultaneously assessed, with a higher speed of analysis. These are the reasons why is the perfect tool for obtaining the maximum data, in the most cost effectiveness way, from hemogram. In fact, there are already published studies with ML in CMD for the detection of infections.^5^, ^29^ Bigorra et al.^5^ have developed an algorithm from hemogram parameters with supervised ML, obtaining a model that is able to classify viral infections or lymphocytic chronic leukaemia with an overall accuracy that ranged from 96% to 98%. Regarding the COVID‐19 disease, Vasse et al. using random forest classifier have obtained an algorithm for diagnosis of COVID‐19 using CPDs,^[^ ^28^ ^]^ and there is only one study with a similar design that the present study, using ML for distinguishing COVID‐19 from flu, but still with a poor number of patients.^30^ Therefore, our study is the first one which has built and algorithm with great accuracy for COVID‐19 diagnosis, that could be available in all clinical centers because it is an inexpensive technique, and can be carried out in less than 1 h. Sensitivity a specificity of diagnostic tests for SARS‐CoV‐2 varied considerably between studies. Molecular test RT‐PCR testing showed the highest performance with 100% PPV and 97.4% NPV, whereas serological testing had lower PPV (84%) and NPV (82.3%), with the sensitivity of both tests worsening in asymptomatic patients.^30^, ^31^ Our algorithm had a better NPV than serological testing (94% vs. 82.3%), indicating that it could replace antigen screening for COVID‐19 in the Emergency Department.

Early in the pandemic, we observed that many patients clinically suspected of COVID‐19 had a negative RT‐PCR, but our model classified them as COVID‐19 positive. These patients were excluded from the database because of the lack of confirmed diagnosis.

Our algorithm was trained during the period of high prevalence of SARS‐CoV‐2 infection (50%), and so we have no evidence of the effectiveness of this model in less prevalent populations. However, we consider that it could be useful as a screening tool and may save RT‐PCR performance or antigen tests in future scenes.

It is important to analyse if this prediction model is able to distinguish SARS‐CoV‐2 infection from other respiratory virus infections with a similar clinical spectrum. Probably flu is the most common infection that may have a clinical overlap with COVID‐19, that is the reason of why we explore the accuracy in a little proportion of patients with flu. The results are quite promising (AUC: 0.89) nevertheless we require more analyses that include a large number of patients to confirm these results.

The main limitation of the present study is that it did not include asymptomatic patients, so we could not evaluate the accuracy of the model in those patients. However, when we studied the accuracy based on clinical severity, the algorithm seemed to perform better in critically ill patients, with significant differences found in hospitalized patients. It is important to note the accuracy of prediction in patients admitted to CCU, albeit not significant. This might be due to the small number of patients included in this group. It will be important to test the algorithm in asymptomatic patients, to realize its potential as a screening tool.

Other main limitation of these study is that all the data came from the same center. For the validation of this model, it is so important to test the algorithm in a different population, so we are requesting for the collaboration with other centers so that we can present results of a prospective study. Regarding to the study population, we have included all type or races, given our influence area includes a multiracial community area. Although for the moment we are not able to collect this data, neither to know if there are differences in the performing of algorithm attending to race. Regarding gender, similarly we presupposed that there should not be differences in cellular changes, and algorithm should rule out in the same way between genders, but this has not been tested. Excluding paediatric patients, age was one of the variables included for the training of the algorithm, we have observed that age has a little importance in isolation for the development of the algorithm. Pending validation in paediatric patients.

Furthermore, it will be interesting to check the model on different subgroups attending to other additional clinical and non‐clinical features such as gender, race or comorbidities.

## 5 CONCLUSION

Our results suggest that ML can be used successfully to generate an algorithm based on hemogram parameters for the diagnosis for COVID‐19 disease, which is applicable to any population and with a global accuracy similar to the gold standard test. This is a great advance for early diagnostic orientation and for guiding clinical decision‐making.

## AUTHOR CONTRIBUTIONS

**S. Gómez‐Rojas:** Investigation (equal); methodology (equal); project administration (equal); writing – original draft (equal); writing – review and editing (equal). **G. Pérez Segura:** Formal analysis (equal); investigation (equal); writing – original draft (equal); writing – review and editing (equal). **J. Ollé:** Formal analysis (lead). **G. Carreño Gómez‐Tarragona:** Writing – review and editing (supporting). **J. González Medina:** Writing – review and editing (supporting). **J. M. Aguado:** Funding acquisition (supporting); writing – review and editing (supporting). **E. Vera Guerrero:** Writing – review and editing (supporting). **M. Poza Santaella:** Writing – review and editing (supporting). **Joaquín Martínez‐López:** Project administration (supporting); supervision (equal); writing – original draft (supporting); writing – review and editing (supporting).

## CONFLICT OF INTEREST STATEMENT

Beckman Coulter S.A® has participated in part of the funding of these research study. There are no other conflicts of interest.

## Supporting information


Appendix S1

## Data Availability

The data that support the findings of this study are openly available in Wiley at https://doi.org/10.1111/jcmm.17864, reference number 17864 Data available on reasonable request from authors.
