# Supplementary material for: A machine learning tool for the diagnosis of SARS‐CoV‐2 infection from hemogram parameters
Source: J Cell Mol Med. 2023 Oct 26;27(22):3423–30. doi: 10.1111/jcmm.17864 (PMC10660618; doi:10.1111/jcmm.17864)
Supplement: Supplementary file 1 — Appendix S1 [file JCMM-27-3423-s001.docx]

**APPENDIX 1**

Meaning of parameters from figure 3.

IWOP: white blood cells differential optical channel.

SDVMO: Monocytes volume standard deviation.

LEU: Total leucocytes.

NEU: Total neutrophils.

MN2MO: Monocyte axial light loss mean.

SDUMO: Monocyte upper median angle light scatter standard deviation.

MNVMO: Monocytes volume mean.

SDLAN: Neutrophils low angle light scatter  standard deviation.

MON: Total monocytes.

SDVLY: Lymphocytes volume standard deviation.

SDMNE: Neutrophils median angle light scatter standard deviation.

MNMNE: Neutrophils median angle light scatter mean.

SDMMO: Monocytes median angle light scatter standard deviation.

MDW: Monocyte distribution width.

SDULY: Lymphocytes upper median angle light scatter standard deviation.

MNUNE: Neutrophils upper angle light scatter mean.

MNULY: Lymphocyte  upper median angle light scatter mean.

SDLAM: Monocytes lower median angle light scatter standard deviation.

SDLNE: Neutrophils lower median angle light scatter standard deviation.

MNMLY: Lymphocytes lower median angle light scatter mean.

SDLMO: Monocytes lower median angle light scatter standard deviation.

MN2LY: Lymphocyte axial light loss mean.

MNLNE: Neutrophils lower median angle light scatter mean.

SDMLY: Lymphocytes median angle light scatter standard deviation.

SDUNE: Neutrophils upper median angle light scatter standard deviation.

SDVNE: Neutrophils volume standard deviation.

LIN: Total lymphocytes.

SD2MO: Monocytes  axial light loss standard deviation.

SDLLY: Lymphocytes lower median angle light scatter standard deviation.

SDCNE: Neutrophils conductivity standard deviation.

MNLLY:  Lymphocytes median angle light scatter mean.

MNCMO: Monocytes conductivity mean.

MNCLY: Lymphocytes conductivity mean.

SDCMO: Monocytes conductivity standard deviation.

SD2NE: Neutrophil  axial light loss standard deviation.

SDCLY: Lymphocytes conductivity standard deviation.

MNLMO: Monocytes lower median angle light scatter mean.

MN2NE: Neutrophil axial light loss mean.

SD2LY: Lymphocyte  axial light loss standard deviation.

MNUMO: Monocytes upper median angle light scatter mean..

MNCNE: Neutrophils conductivity mean.

MNVNE: Neutrophils volume mean.

MNVLY: Lymphocytes volumen mean.

MNLAM: Monocyte low angle light scatter mean.

SDLAL: Lymphocyte lower median angle light scatter standard deviation.

IEGC: Early granulocytic cells.

MNLAN: Neutrophils low angle light scatter mean.

MNMMO: Monocytes median angle light scatter mean

MNLAL: Lymphocytes low angle light scatter mean.
